# Traditional Chinese herb for low endometrial receptivity and its effect on pregnancy: Protocol for a systematic review and meta-analysis

**DOI:** 10.1097/MD.0000000000017841

**Published:** 2019-11-22

**Authors:** Mei Jiang, Ling Huang, Xiaohong Gu, Tiegang Liu, Jia Kang, Ting Wang

**Affiliations:** a Beijing University of Chinese Medicine; b Gulou Hospital, Affiliated hospital of Capital Medical University, Beijing, China.

**Keywords:** low endometrial receptivity, protocol, systematic review, traditional Chinese herbs

## Abstract

**Background:**

Low endometrial receptivity is associated with infertility in women through multiple and complex mechanisms. Existing treatments are not always effective. Symptomatic drugs such as estradiol valerate and/or aspirin do not completely solve the problem. Traditional Chinese herbs have been widely used in infertility and uterine disease including low endometrial receptivity. However, their effectiveness and safety are still obscure and deserve further investigation.

**Objective:**

To assess the effect and safety of traditional Chinese herbs in treating low endometrial receptivity.

**Methods:**

We will summarize and meta-analyze randomized controlled trials (RCTs) of traditional Chinese herbs for the treatment of low endometrial receptivity. RCTs comparing traditional Chinese herbs with blank control, placebo, or conventional therapies will be included. RCTs comparing traditional Chinese herbs plus conventional therapies with conventional therapies alone will also be included. The following electronic databases will be searched: PubMed, Cochrane Library, EMBASE, CNKI, CBM, VIP, and WANFANG DATA. The methodological quality of RCTs will be assessed using the Cochrane risk assessment tool. All trials included will be analyzed according to the criteria of the Cochrane Handbook. Review Manager 5.3, R-3.5.1 software will be used for publication bias analysis. GRADE pro GDT web solution will be used for evidence evaluation.

**Results:**

This review will evaluate the effects of traditional Chinese herbs on estradiol, progesterone, thickness, volume, and perfusion index(PI) of the endometrium, pregnancy rate, and symptoms.

**Conclusions:**

This review will provide clear evidence to assess the effectiveness and safety of traditional Chinese herbs for low endometrial receptivity.

**OSF registration number:**

DOI 10.17605/OSF.IO/M85VT.

## 1 Introduction

The application of assisted reproductive technology(ART) has increased significantly since the first in vitro fertilization and embryo transfer (IVF-ET) conceived child was born in 1978, which contributes to the success of 5 million births worldwide.^[1]^ With the development of ART, how to improve the embryo implantation rate has become a research hotspot. However, some failure in ART has been identified, which is related to repeated implantation failure (RIF). RIF was mainly due to low endometrial receptivity.^[2]^

Endometrial receptivity refers to the receptivity of the endometrium to the embryo. During ovulation period, patients can be defined as low receptivity according to the endometrium thickness under 8 mm and the endometrial PI under 2.^[3,4]^ Endometrial receptivity for the embryo is established and maintained through a series of precise cellular and molecular events. With low endometrial receptivity, the balance of signaling pathways and gene expression programs was broken to drive an abnormal menstrual cycle and reduce an embryo-receptive state to allow implantation during the window of receptivity.^[5]^ In recent years, with the spread of clinical guidelines and increased access to drugs, RIF has been reduced. Although the currently available drugs have achieved widespread success, there have been no strategies to cure. Existing treatments are not always effective.^[6,7]^ Symptomatic drugs such as estradiol valerate and/or aspirin, which increase the thickness and volume of the endometrium, do not completely solve the problem.^[8,9]^ More unconventional therapies should be valued. Acupuncture are able to restore fertility in most cases by method of promoting blood circulation and removing blood-stasis, which may affect both ovarian and endometrium functions, with a final alteration in oocyte maturation and endometrial epithelium receptivity.^[10,11]^ We want to know if traditional Chinese herbs also have an effect on low endometrial receptivity. We decided to conduct this study after a systematic search but no similar study was founded. This systematic review has been registered on OSF (DOI 10.17605/OSF.IO/M85VT).

## 2 Methods

This systematic review has been registered in OSF (https://osf.io/m85vt/), registration number: DOI 10.17605/OSF.IO/M85VT. Systematic review is a secondary literature research that does not require direct contact with patients, so the ethical approval and patient consent form are not necessary. We will develop and report this study in compliance with the Preferred Reporting Items for Systematic Reviews and Meta-Analyses (PRISMA).^[12]^ The procedure of this protocol will be based on PRISMA-P guidance.^[13]^

### 2.1 Database search

Three English medical databases (Cochrane Library, PubMed, and EMBASE) and 4 Chinese medical databases (China National Knowledge Infrastructure Database (CNKI), Chinese Biomedical Literature Database (CBM), VIP Chinese Science and Technology Periodical Database (VIP), and Wan Fang Data) will be systematically searched from their inceptions up to September 10, 2019. The search strategy will be based on the guidance of the Cochrane handbook.^[14]^ The search formulas of the databases are adjusted according to the following forms: (traditional Chinese herbs OR herbs OR herb OR prescription OR formula OR potion OR powder) AND (low endometrial receptivity OR endometrial thickness OR low uterine receptivity) AND (random∗). All relevant publications including academic dissertation and conference will be researched. There will be no language and publication date restrictions.

### 2.2 Inclusion criteria

#### 2.2.1 Types of studies

Only randomized controlled trials (RCTs) will be included.

#### 2.2.2 Types of participants

All of the participants who were diagnosed as low endometrial receptivity.

#### 2.2.3 Types of intervention

Traditional Chinese herbs as the intervention treatment compared with blank control, placebo or conventional treatment will be selected. Traditional Chinese herbs in combination with conventional therapies compared with conventional therapies alone will also be included. All interventions will be treated for no less than 12 weeks.

#### 2.2.4 Types of outcome measures

Primary Outcomes: estradiol, progesterone, endometrial thickness, endometrial volume, perfusion index(PI) or resistance index(RI) of the endometrium, and pregnancy rate.

Secondary Outcomes: low endometrial receptivity exacerbations, QOL and adverse event. The time endpoint of the above outcomes will be no earlier than 12 weeks after starting the medication.

### 2.3 Exclusion criteria

1. The unrelated and duplicated documents will be deleted.
2. Animal experiments, reviews, theoretical discussions, experience summaries, and case reports.
3. Review articles without original data.

### 2.4 Data collection and extraction

Referring to the Cochrane collaborative network system evaluator handbook:^[13]^

(1) Importing the search results into the document management software of NoteExpress (version:3.2, Beijing Aegean Software Company, Beijing, China);
(2) Excluding the duplicate literature using NoteExpress3.2 and excluding the unrelated articles by reading the title and abstract;
(3) Reading the full text and reserving clinical studies that meet the inclusion criteria.

Two researchers (MJ and LH) will extract the data independently using a self-developed data extraction form. The differences encountered in the process will be resolved by discussing with another team member (JK), to determine, by agreement, the final selection of studies.

Data extraction contents will include:

(1) General information: research ID, author, title, publication status, report sources and fund support.
(2) Methodology information: design, number of arms, random sequence generation, allocation concealment, blinding, incomplete outcome data, selective reporting, sample size calculation, and baseline comparability.
(3) Participant information: diagnostic criteria, inclusion criteria, exclusion criteria, setting, population, sample size, age, gender, and course of disease.
(4) Intervention information: name of intervention and comparison, dosage form, comparison, duration of treatment, and patient follow-up.
(5) Outcomes.
(6) Adverse events.

**Figure 1:**
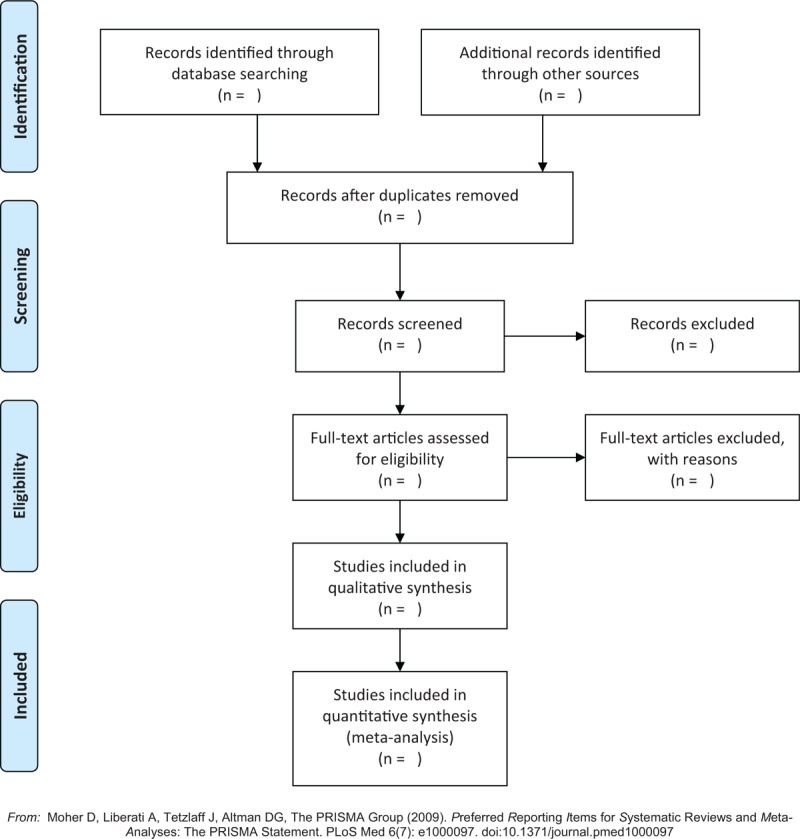
Flow chart of the selection process.

The selection process was showed in a PRISMA flow chart (http://www.prisma-statement.org/)^[14]^ (Fig. 1).

### 2.5 Assessment of methodological quality

Risk of bias will be assessed by Cochrane risk assessment tool^[13,14]^ in 7 domins: random sequence generation, allocation concealment, blinding of the participants and personnel, blinding of outcomes assessment, incomplete outcome date, selective outcome reporting, and other bias. These domains will classify “low risk” if adequate, “high risk” if not adequate and “Unclear” if not well described by the authors in such a way that its adequacy is describable.

The 2 researchers (MJ and LH) will independently assess the risk of bias for each included study. We will use “L,” “H,” and “U” as a code for the evaluations of the above bias risks. “L” indicating a low risk of bias, “H” indicating a high risk of bias, “U” indicating that the risk of bias is unclear. Disagreements will be resolved by discussion between all the researchers. When necessary, we will contact the study authors to inquire some missing information. Trials of high risk of bias will be considered when conducting sensitive analysis.

### 2.6 Data synthesis and analysis

Review Manager Software (RevMan, Version 5.3 for windows, The Cochrane Collaboration, Oxford, England) will be used to analyze and synthesize the outcomes. Quantitative synthesis will be done when clinical heterogeneity is not considered by at least 2 authors in discussion. Continuous variable will be described by mean difference (MD), *P* value and 95% confidence interval (CI). For dichotomous outcomes, we will use the relative risk (RR), with 95% CI and *P* values, to evaluate the efficacy and safety of traditional Chinese herbs. *I*^2^ test will be used to judge the heterogeneity of meta-analysis. *I*^2^ value > 50% or more will be considered as an indication of substantial heterogeneity. If heterogeneity exists in the pooled studies, the data will be analyzed using a random effects model. Otherwise, a fixed effect model will be adopted. Sensitivity analysis or subgroup analysis will be performed if included trials are sufficient. The grouping factor for subgroup analysis will be age, low endometrial receptivity severity and treatment duration. Qualitative description will be adopted if clinical heterogeneity exists.

### 2.7 Publication bias

The publication bias will be analyzed by the Egger test. The analysis software is *R* 3.5.1 for Windows.

### 2.8 Quality of evidence

This study evaluates the evidence according to GRADE standard, which refers grading of recommendations assessment, development and evaluation.^[15]^ GRADE Pro GDT online software will be used to form the summary of findings table (SoF table).

## 3 Discussion

Traditional Chinese herbs, used for treating and preventing several diseases has had been assessed systematically in recent years. Among them, infertility have got a lot of attention. For infertile patients, IVF-ET is reserved as boon in which other methods such as fertility drugs, surgery, and artificial insemination have not worked, and currently be regarded as the most effective and reliable method. Whereas, IVF-ET is a complex and expensive procedure, that limits their range of applications. The More unconventional therapies should be valued to increasing the success rate of IVF-ET is crucial for treating female infertility. Endometrial receptivity plays a role in the development of IVF-ET because of its function to embryo implantation. Traditional Chinese herbs are able to restore fertility in most cases, which may affect both ovarian and endometrium functions, with a final alteration in oocyte maturation and endometrial epithelium receptivity. Are traditional Chinese herbs effective and safe for low endometrial receptivity? In order to answer this question, we searched databases but found no systematic review of RCTs published. We need more comprehensive and credible evidence to guide clinical practice. We should also assess the shortcomings of existing clinical evidence to guide future clinical trials. This study will solve the above problems.

## Author contributions

Ling Huang and Mei Jiang conceived and designed the project. Ling Huang, Mei Jiang, Xiaohong Gu, Tiegang Liu, Jia Kang and Ting Wang implemented the methods. Jia Kang contributed analysis tools and edited review. Tiegang Liu contributed reagents/materials. Xiaohong Gu and Ting Wang revised the manuscript. All authors read and approved the final manuscript. Mei Jiang and Ling Huang contributed equally to this work.

## References

[1] Kikuchi K Shibahara H Hirano Y Antinuclear antibody reduces the pregnancy rate in the first ivf-et treatment cycle but not the cumulative pregnancy rate without specific medication. Am J Reprod Immunol 20;50:363–7. 14672342 10.1034/j.1600-0897.2003.00088.x

[2] Fukui Y Hirota Y Matsuo M Uterine receptivity, embryo attachment, and embryo invasion: multistep processes in embryo implantation. Reprod Med Biol 2019;18:10.1002/rmb2.12280 PMC6613011 31312101

[3] Sudoma I Goncharova Y Zukin V Optimization of cryocycles by using pinopode detection in patients with multiple implantation failure: preliminary report. Reprod Biomed Online 2011;22:590–6. 21493157 10.1016/j.rbmo.2011.02.004

[4] Alcázar Juan Luis Mercé Endometrial volume and vascularity measurements by transvaginal 3-dimensional ultrasonography and power doppler angiography in stimulated and tumoral endometria. J Ultrasound Med 2005;24:1091–8. 16040824 10.7863/jum.2005.24.8.1091

[5] Blesa D Ruiz-Alonso Maria Clinical management of endometrial receptivity. Semin Reprod MedV 32 2014;410–4. 10.1055/s-0034-1376360 24959823

[6] Haas J Zilberberg E Nahum R Does double trigger (gnrh-agonist + hcg) improve outcome in poor responders undergoing ivf-et cycle? A pilot study. Gynecol Endocrinol 2019;1–3. 10.1080/09513590.2019.1576621 30810400

[7] Kasum M Kurdija K Ore?Kovi? S Combined ovulation triggering with gnrh agonist and hcg in ivf patients. Gynecol Endocrinol 2016;1–5. 10.1080/09513590.2016.1193141 27275861

[8] Fatemi HM Popovic-Todorovic B Implantation in assisted reproduction: a look at endometrial receptivity. Reprod Biomed Online V 27 2013;530–8. 10.1016/j.rbmo.2013.05.018 23933035

[9] Margalioth EJ Ben-Chetrit A Gal M Investigation and treatment of repeated implantation failure following ivf-et. Hum Reprod V 21 2006;3036–43. 10.1093/humrep/del305 16905766

[10] Zhong Y Zeng F Liu W Acupuncture in improving endometrial receptivity: a systematic review and meta-analysis. BMC Complement Altern Med 2019;19:10.1186/s12906-019-2472-1 PMC6417024 30866920

[11] Shuai Z Lian F Li P Effect of transcutaneous electrical acupuncture point stimulation on endometrial receptivity in women undergoing frozen-thawed embryo transfer: a single-blind prospective randomised controlled trial. Acupunct Med 2015;33:9–15. 25303950 10.1136/acupmed-2014-010572

[12] Re: preferred reporting items for systematic review and meta-analysis protocols (prisma-p) 2015: elaboration and explanation. BMJ 2018. 10.1136/bmj.g7647 25555855

[13] Higgins JPT Thomas J Chandler J *Cochrane Handbook for Systematic Reviews of Interventions version 6.0* (updated July 2019). Cochrane, 2019. Available from www.training.cochrane.org/handbook.

[14] Liberati A Altman DG Tetzlaff J The prisma statement for reporting systematic reviews and meta-analyses of studies that evaluate health care interventions: explanation and elaboration. Epidemiol Biostat Public Health 2009. 10.1016/j.jclinepi.2009.06.006 19631507

[15] Shamseer L Moher D Clarke M Preferred reporting items for systematic review and meta-analysis protocols (prisma-p) 2015: elaboration and explanation. BMJ 2015;349:g7647–17647. 10.1136/bmj.g7647 25555855

